# Locking plate versus external fixation in the treatment of displaced femoral supracondylar fracture in children

**DOI:** 10.1186/s13018-020-01759-7

**Published:** 2020-06-23

**Authors:** Jin Li, Xikai Guo, Hai Qiang Wang, Changjie Yue, Kailei Chen, Jiewen Ma, Jing Wang, Xin Tang

**Affiliations:** 1 Department of Orthopaedic Surgery, Union Hospital, Tongji Medical College, Huazhong University of Science and Technology, Wuhan, 430022 China; 2 Tongji Medical College, Huazhong University of Science and Technology, Wuhan, 430030 China; 3 Institute of Integrative Medicine, Shaanxi University of Chinese Medicine, Xixian Avenue, Xixian District, Xi’an, 712000 China; 4 Department of Radiology, Union Hospital, Tongji Medical College, Huazhong University of Science and Technology, Wuhan, 430022 China

**Keywords:** Locking plate, External fixation, Supracondylar femoral fractures, Pediatric fracture

## Abstract

**Background:**

Displaced supracondylar femoral fractures (SFF) are difficult injuries to treat in children. Several techniques have been widely used but few studies have compared the merits and drawbacks of each surgical intervention in order to analyze clinical values. The aim of this study was to (1) evaluate postoperative and functional conditions after treatments with locking plate (LP) or external fixation (EF), (2) observe adverse events associated with these two techniques, and (3) evaluate the clinical value of these two techniques.

**Methods:**

Twenty-eight patients less than 14 years of age were included in this study with supracondylar femoral fractures. They underwent locking plate or external fixation in authors’ hospital. The postoperative healing and functional outcome were elevated according to radiographic and clinical measures, including American Knee Society Score (KSS). Fisher’s exact test and independent samples *t* test were used for statistical analysis.

**Results:**

All fractures healed without delayed union. The KSS scoring results of locking plate and external fixation groups were both excellent. The alignment of lower limbs was acceptable with knee valgus less than 2° for all involved patients. In addition, leg length discrepancy was less than 1 cm. No acute or severe complications were noted. There was significant difference in union time (*p* = 0.03), operating time (*p*< 0.001), intraoperative blood loss (*p*< 0.001), and limb length discrepancy (*p* = 0.04) between LP group and EF group.

**Conclusions:**

External fixation is superior than locking plate in terms of union, operation time phrases, and intraoperative blood loss. EF techniques are better options for treating displaced supracondylar femoral fracture in children.

**Level of evidence:**

Retrospective comparative study; level III.

## Background

Supracondylar femoral fracture (SFF) is a rare kind of injury [1]. In clinical practice, SFF is also defined as a fracture box, given that the distance between the fracture center and the knee is equivalent to or less than the widest part of the width of both femoral condyles [2]. Compared to femoral shaft fractures accounting for 1.6% of pediatric fractures [3], distal femoral fractures only comprise 0.4% of all types of fractures [4]. Nicholas et al. reported the incidence rate of SFF as 12% in pediatric femoral fractures [5]. Notwithstanding SFF is uncommon, it has severe impact on children’s health and welfare, with detrimental consequences consisting of the shortening and premature arrest of growth, angular deformity, joint motion compromising, ligamentous laxity, and thigh atroph y[6].

Supracondylar femoral fracture can be simply divided into non-displaced and displaced types. Non-displaced fractures can usually be treated by conservative treatment, but ultimately resulting in a high risk of adhesion and subsequent stiffness [7]. Displaced SFF should undergo surgical procedures, including external fixation, fixed-angle device, plate fixation, intramedullary nailing, arthroplasty, and distal femoral replacement [8]. Though these surgical interventions offer pediatric orthopedists many choices, the optimal method of treatment for SFF in children remained largely ambiguous. Pediatric patients have to endure substantially in hospitalization, school-leaving time, perioperative pain, and reduced activity for weeks or even months [9]. This study aimed to evaluate the clinical value of locking plate versus external fixation in the treatment of children’s SFFs.

## Methods

The medical charts were reviewed for collection of information below, gender, age, height, weight, fractured limb, mechanism of injury, associated injuries, and fracture types (transverse type, oblique type, or comminuted type). From April 2010 to August 2017, patients treated in authors’ institution satisfying the following criteria were recruited in this study: (1) age < 14 years, (2) diagnosis as SFF confirmed on radiographs (AO/OTA classification for distal femoral fracture 33A or C), (3) undergoing surgical procedures with locking plate or external fixation, and (4) follow-up until complete clinical and radiographic union or at least 24 months. Exclusion criteria were designed as followed: (1) follow-up less than 24 months, (2) distal femoral fractures involving intra-articular fractures, knee ligament tears, and neuro or vascular injury, (3) congenital anomalies of femur or old fractures of femur (> 4 weeks), (4) preexisting significant ipsilateral limb diseases or comorbidities hindering recovery from fractures, (5) pathologic SFFs, and (6) incomplete medical records. This retrospective study was approved by the ethical review board in author’s institutional, and all the patients’ parents gave their informed consent for the method of fixations which they would accept after being explained the pros and cons and the potential complications of each method.

**Fig. 1:**
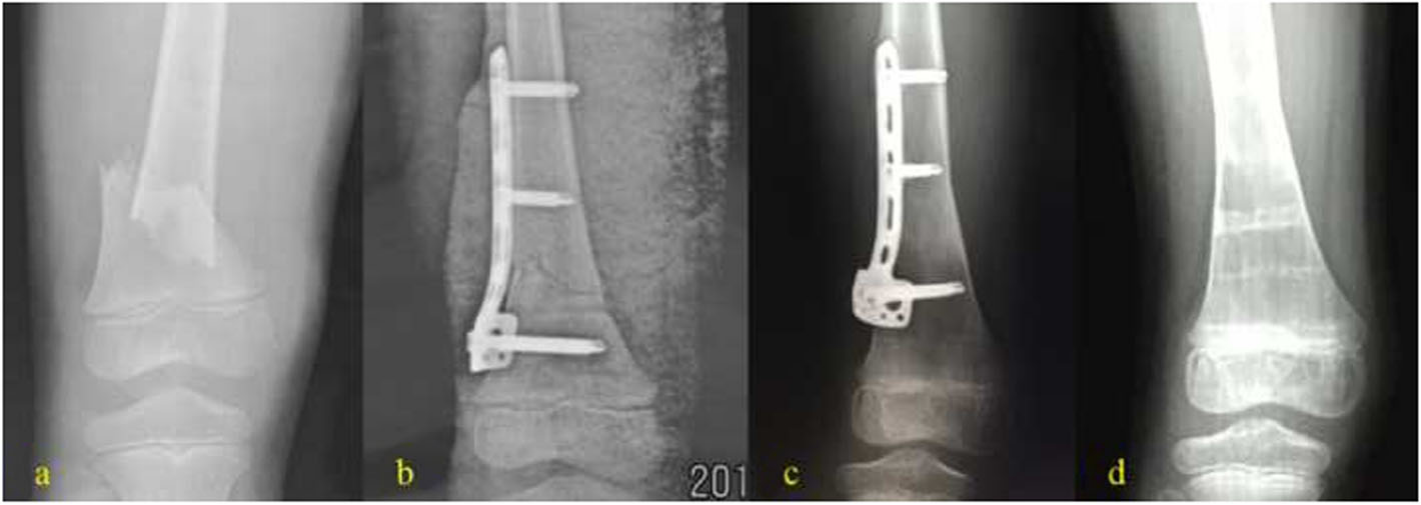
Anteroposterior radiographs showing pediatric displaced supracondylar femoral fractures treated with locking plate. male, 6 years old, right side. **a** Pre-operation. **b** Post-operation. **c** 11 months after surgery. **d** 18 months after surgery

**Fig. 2:**
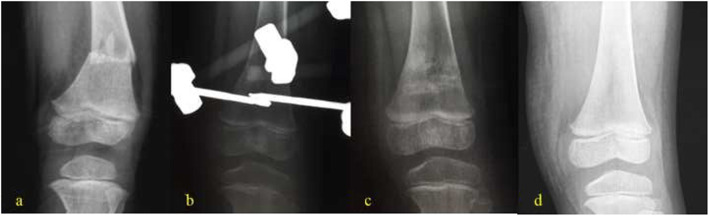
Anteroposterior radiographs showing pediatric displaced supracondylar femoral fractures treated with external fixation. female, 4 years old, left side. **a** Pre-operation. **b** Post-operation. **c** 2 months after surgery. **d** 18 months after surgery

In the LP group [10–15], open reduction of femoral condyles was achieved by traction after general anesthesia. The beveled tip of the plate allows minimally traumatic insertion among submuscular structures, the muscles vastus laterals, and periosteum. The plate should be parallel to the lateral cortex in front, centered on the femoral diaphysis in profile, and the distance between the end of plate and the epiphyseal line of the distal femoral was 1 to 2 cm at least. The reduction and instrumentation were confirmed under fluoroscopy. Finally, at least three screws were utilized for construction in each main fragment, and suction drains were placed before wound closure (Fig. 1). In the EF group, traction reduction was performed on the orthopedic traction bed with C-arm x-ray fluoroscopy after general anesthesia. During the process, displacements were corrected in proper order as the overlapping, lateral and angular, and the rotation and separation deformities, ensuring rotation and overlap deformity corrected, fracture ends restored satisfactorily. Five open fracture cases were reduced after wound debridement. External fixation pins with a diameter of 3.5 to 4.5 mm was inserted along the guide needle path and penetrate bilateral cortex. The pins insertion site should avoid important blood vessels, nerves, and tendons. It must be noted that the distance between the external fixation pins and the epiphyseal line of the distal femoral was about 1 to 2 cm, and external fixation pins should be inserted about 2 cm away from the fracture end. Two external fixator pins 3.5 to 4.5 mm in diameter were placed uniplanarly in the lateral part of the middle femoral shaft. Under x-ray fluoroscopy, the external fixator was connected as a designed frame of quadrilateral and avoid knee spanning after proper position of the external fixator, and all the fixing nuts were tightened. (Fig. 2).

In both groups, an above knee backslab was applied with operated lower limb in 30° of flexion position for the management of swelling and pain. Penicillin antibiotics were used for 3–5 days according to the wound infection in the exposed fractures. Between 2 and 3 weeks, backslabs were removed according to the swelling and pain relief by patients’ parents, and gentle knee mobilization is allowed. Rehabilitation exercises were performed at home with given instructions. Four weeks after operation, patients were encouraged to stand with no weight-bearing. The time to start weight-bearing exercises was decided according to the AO/OTA types of SFFs, the type of fixation, and the results of clinical x-ray follow-up. Radiographic union is defined as the osteotomy gap that is no longer visible in three of the four cortices on antero-posterior and lateral images. Generally, weight-bearing exercises begin 10 to 12 weeks after operation. During the follow-up period, clinical measures were recorded using a spreadsheet, including demographic characteristics, duration for union, time of follow-up, and post-operative and functional status. As well, data were collected from medical records, i.e., traumatic limb sides, AO/OTA classification, fracture types, cause of fracture, length of stay in hospital, and operation-related information. Postoperative healing and functional recovery were evaluated according to the results of radiographic and clinical examination. When the three cortices of the femur were united on the anteroposterior (AP) and lateral views, radiographic union could be identified. Clinical healing is defined as the absence of pain, whether bearing weight or applying pressure to examine the injured area [16].

The SPSS 22.0 (IBM SPSS Statistics, the USA) software was used to perform statistical analyses. A *p* value of less than 0.05 was considered statistically significant. Fisher’s exact test indicated no statistical difference between two groups in terms of fractured limb (*p* = 1), fractured type (*p* = 0.4), cause of injury (*p* = 0.8), and AO classification (*p* = 0.4).

## Results

According to the inclusion and exclusion criteria, we identified a total of 28 children, 13 managed with locking plate and 15 with external fixation. There were 7 boys and 6 girls in the LP group with a mean ± SD age of 10.1 ± 2.3 years (range, 6.0–14.0 years) and 6 boys and 9 girls in the EF group with a mean ± SD age of 7.6 ± 2.3 years (range, 3.9–11.8 years) (Table 1). The AO/OTA classification of patients in the LP group was 33A1 or 33A2 or 33A3, and all the patients in the EF group were 33A except for an 11.8-year-old girl whose AO/OTA classification was 33C1 (Table 1). All patients in the LP group were closed fractures. In the external fixation group, there were five cases with open fractures and 10 cases with closed fractures. All five open fracture were Gustilo and Anderson I type, got one issue of debridement and suture in emergency surgery. None had neurovascular impairment. The follow-up time was 54.8 ± 28.8 (range 24–112) months for the LP group and 33.9 ± 7.1 (range 24–50) months for the EF group. External fixator in the EF group was removed after clinical and radiological evaluation at week five to eight followed. Plate in the LP group was removed with general anesthesia at 6 to 12 months followed. There were no healing deformities, deep infection after surgical intervention, or symptoms requiring further treatment in all the patients. The absence of infection in this study may be attributed to the effectiveness of antibiotic therapy. Only two patients in the external fixator group had superficial skin infection on needle path, which disappeared after nursing and antibiotics applying. None of the study subjects had neurovascular impairment.

**Table 1:** Characteristics of pediatric patients with supracondylar femoral fractures treated with locking plates and external fixations

Variable	Locking plate	External fixation	p*
Age (year)	10.1 ± 2.3	7.6 ± 2.3	0.009
Fractured limb (number of patients)			1.000
Left	7 (53.8%)	9 (60.0%)	
Right	6 (46.2%)	6 (40.0%)	
Fractured type (number of patients)			0.448
Transverse	3 (23.1%)	7 (46.7%)	
Oblique/spiral	4 (30.8%)	3 (20.0%)	
Comminuted	6 (46.1%)	5 (33.3%)	
Cause of injury (number of patients)			0.841
Fall	8 (61.5%)	7 (46.7%)	
Vehicle accident	5 (38.5%)	7 (46.7%)	
Other	0 (0.0%)	1 (6.6%)	
AO classification			0.393
33A1	3 (23.1%)	7 (46.7%)	
33A2	4 (30.8%)	3 (20.0%)	
33A3	6 (46.1%)	4 (26.7%)	
33C1	0 (0.0%)	1 (6.6%)	
Open/close fracture			0.044
Open	0 (0.0%)	5 (33.3%)	
Close	13 (100.0%)	10 (66.7%)	
Follow-up time (month)	54.8 ± 28.8 (24.0–112.0)	33.9 ± 7.1 (24.0–50.0)	0.024

*Fisher’s exact test

All patients achieved radiographic union after surgical intervention with locking plate or external fixation (Figs. 1 and 2). The radiological union time was 13.6 ± 1.5(range12–16) weeks for the LP group and 12.2 ± 1.7(range 10–16) weeks for the EF group. The knee flexion range of motion was 127.7 ± 7.3° (range 120–140) for locking plate group and 124.0 ± 4.7° (range 120–135) for external fixation group. The knee extension range of motion was 3.8 ± 2.2° (range 0–5) for the locking plate group and 3.3 ± 2.4° for the external fixation group (range 0–5). The KSS postoperative score of locking plate group was 96.4 ± 2.3 (range 94–99), and the KSS functional score of this group was all full score. The KSS postoperative score of external fixation group was 97.5 ± 2.6 (range 93–100), and the KSS functional score was 90 in 5 cases and 100 in the other 10 cases. There was no significant difference in statistics for range of motion for flexion (*p* = 0.1), range of motion for extension (*p* = 0.6), and KSS postoperative score (*p* = 0.2) except for union time (*p* = 0.027) between the LP group and EF group (Table 2).

**Table 2:** Operation related information and post-operation evaluations

	Groups		
Variables	Locking plate	External fixation	p*
Operating duration (min)	88 ± 9.9	49.2 ± 20.5	< 0.001
Preoperative stay (hour)	59.4 ± 61.2	70.5 ± 82.9	0.687
Intraoperative blood loss (ml)	50.4 ± 11.3	18.0 ± 20.9	< 0.001
Total days in hospital (day)	12.8 ± 2.8	9.9 ± 5.4	0.081
Time to union (week)	13.6 ± 1.5	12.2 ± 1.7	0.027
Limb length discrepancy (cm)	0.37 ± 0.14	0.50 ± 0.17	0.036
KSS postoperative score	96.4 ± 2.3	97.5 ± 2.6	0.226
Valgus deformity degree (°)	1.46 ± 0.52	1.33 ± 0.49	0.700

*Independent samples *t* test

Follow-up evaluation indicated that the angle of knee valgus deformity was less than 2° and the length difference of lower limbs less than 1 cm in both groups, based on KSS post-operative score system. The evaluation results were satisfactory to doctors and patients. There were no acute and/or severe adverse events in both groups. There was significant difference in open/close fracture type (*p* = 0.04), operating time (EF group 49.2 ± 20.5 min; LP group 88 ± 9.9 min; *p*< 0.001), intraoperative blood loss (EF group 18.0 ± 20.9 ml; LP group 50.4 ± 11.3 ml; *p*< 0.001), and limb length discrepancy (*p* = 0.04) between LP group and EF group (Tables 1 and 2). There was no significant difference in statistics for sides of fractured limbs (*p* = 1), fractured types (*p* = 0.5), cause of injury (*p* = 0.8), and AO classification (*p* = 0.4) between LP group and EF group (Tables 1 and 2).

## Discussion

Supracondylar fracture of femur usually occurs in high-energy injuries, often accompanied by severe soft tissue contusion. Fracture ends may be comminuted or displaced and fracture lines extend to the knee joint involving joint surface and knee extension device injuries. The general non-surgical fixation treatment is often difficult to achieve the requirements of complete reduction. Therefore, it will lead to knee stiffness, joint range of motion reduced, walking difficulties, feeling of pain, or other functional disorders sequelae. Surgical intervention in the treatment of SFF can stabilize the fracture site and prevent the displacement of fracture ends so as to reduce complications. There are some differences between the treatment and prognosis of the SFF and that of the femoral shaft fracture [3, 17]. It is very important and necessary to choose appropriate treatment for SFF in children. In view of this, this research assessed post-operative healing, functional recovery, complications and risk factors for developing complications after locking plate, or external fixation treatment in order to figure out an optional method to deal with the SFF in children.

Because incisions were made in the process of locking plate surgeries, bleeding is inevitable more often than external fixation surgeries without incisions. The amount of bleeding during the operation in EF group is significant less than that in LP group (*p*< 0.001). Owing to the incisions were made during the locking plate surgeries, it took a lot of time on sutures of incisions. The operation time of EF group is significantly shorter than that of LP group (*p*< 0.001). Because of the thickening of the osteogenic periosteum, bone healing is usually rapid in children [18]. There was significant difference in statistics for age between two groups (*p* = 0.009). The age of patients directly affects the rate of fracture healing: the younger the children are, the faster the fracture healing is. External fixation will not damage the internal and external periosteum of bone, nor destroy the growth of the original callus without stripping soft tissue, which is more conducive to fracture healing. Therefore, the union time of EF group is significant less than that in LP group (*p* = 0.027).

External fixation showed a number of advantages just as we found above. However, in the final results of range of motion for flexion (*p* = 0.1) or extension (*p* = 0.6) and KSS scores (*p* = 0.2), external fixation failed to show that there were more obvious superiorities compared with locking plate (Table 2). As for locking plate, integration of bone with steel plate and screw can achieve strong internal fixation, which meet the requirements of early functional exercise after operation and is beneficial to the recovery of knee joint function. Although there is no significant difference between the two groups for function and rehabilitation results, it did not mean that we do not recognize the deadly disadvantage of LP. Supracondylar femoral fractures in children are always near phisys. Open reduction with LP fixation is very easy to affect the growth of bone. There was significant difference in statistics in limb length discrepancy between two groups (*p* = 0.04), but the limb length discrepancy of both groups was less than 1 cm (0.37 ± 0.14 cm in LP group and 0.50 ± 0.17 cm in EF group). There were no subjective sensory abnormalities in the range of motion, strength, and appearance of knee joint in this research. The limb length discrepancy in both groups may not affect the normal walking function, motion ability, and stability of children. Orthopaedic surgeons perform LP in SFF must have clear conscious about this risk and operate carefully, so as not to stimulate phisys and affect the growth and shaping of children’s femur. All these lead to longer learning curve and operation time. Because of the reality that there is no specialist pediatric orthopaedic surgeons in most developing country and general orthopaedic surgeons are more familiar with LP technique, they may tend to treat SFF in children with LP.

This study had many limitations besides relatively low numbers of patients and significant difference in age between two groups. The choice of external fixation for open fractures results in selective bias in grouping. For open fractures, the conventional view is that internal fixation such as locking plate is easy to increase the risk of infection, while external fixation is suitable for open fractures [19–22].

## Conclusion

In conclusion, the postoperative conditions and functional conditions after treatments between EF group and LP group were similar, and both groups had excellent assessment results. However, the union time, operation time, and intraoperative blood loss were less for the patients in EF group. LP should be avoided to use for SFF in children unless there were not any other fixator for chose.

## Data Availability

The datasets used and/or analyzed during the current study are available from the corresponding author on reasonable request.

## Abbreviations

SFF: Supracondylar femoral fractures
LP: Locking plate
EF: External fixation
